# A Case of Colchicine Toxicity Presenting With Euglycemic Diabetic Ketoacidosis

**DOI:** 10.7759/cureus.39063

**Published:** 2023-05-15

**Authors:** Sean D Delshad, Angela Kleiber

**Affiliations:** 1 Medicine, David Geffen School of Medicine at the University of California, Los Angeles, Los Angeles, USA

**Keywords:** diabetic keto acidosis, internal medicine (general medicine), dka with normal blood glucose, precipitating factors for euglycemic dka, colchicine poisoning

## Abstract

Colchicine has a narrow therapeutic window and a high risk of toxicity when co-administered with CYP3A4 inhibitors and P-glycoprotein inhibitors. Colchicine toxicity is associated with various metabolic disturbances and can cause multiorgan failure and death. However, to our knowledge, there are no documented reports of colchicine toxicity initially presenting as euglycemic diabetic ketoacidosis (DKA). We present a case of colchicine toxicity with concomitant euglycemic DKA in a man with long-term colchicine use who was also prescribed clarithromycin and dapagliflozin.

## Introduction

Colchicine has long been utilized as a treatment for gout and other inflammatory conditions, including familial Mediterranean fever and pericarditis. Colchicine has a narrow therapeutic window with common adverse effects, including abdominal pain, nausea, vomiting, and diarrhea, which usually resolve with dose adjustments [1]. Colchicine can also cause significant toxicity, resulting in multiorgan failure and even death [2]. Various medications, most notably CYP3A4 inhibitors and P-glycoprotein inhibitors, should not be co-administered with colchicine as they substantially increase the risk of toxicity. The clinical features of colchicine toxicity are well documented in the literature [2-4], but the rarity of colchicine toxicity and its clinical resemblance to a multitude of different pathological processes necessitate physicians to have a high index of suspicion for its diagnosis. Colchicine toxicity is known to cause metabolic acidosis, but, to our knowledge, there are no previous documented cases of colchicine toxicity presenting with euglycemic diabetic ketoacidosis (DKA).

## Case presentation

An 80-year-old man with a history of coronary artery disease, type 2 diabetes mellitus, and gout presented to the emergency room with abdominal pain and distention. Approximately two months prior to this current presentation, the patient suffered from a non-ST elevation myocardial infarction, for which he underwent percutaneous coronary intervention (PCI). Following PCI, the patient was started on dapagliflozin, and after hospital discharge, the patient began to experience abdominal bloating, diarrhea, and decreased appetite. The patient sought care with his primary care physician and was diagnosed with *Helicobacter pylori* infection. He was started on triple therapy with amoxicillin, clarithromycin, and pantoprazole. The patient then experienced worsening diarrhea, followed by constipation and progressively worsening diffuse abdominal pain and distention, for which he presented to the emergency room one week after starting triple therapy.

**Figure 1:**
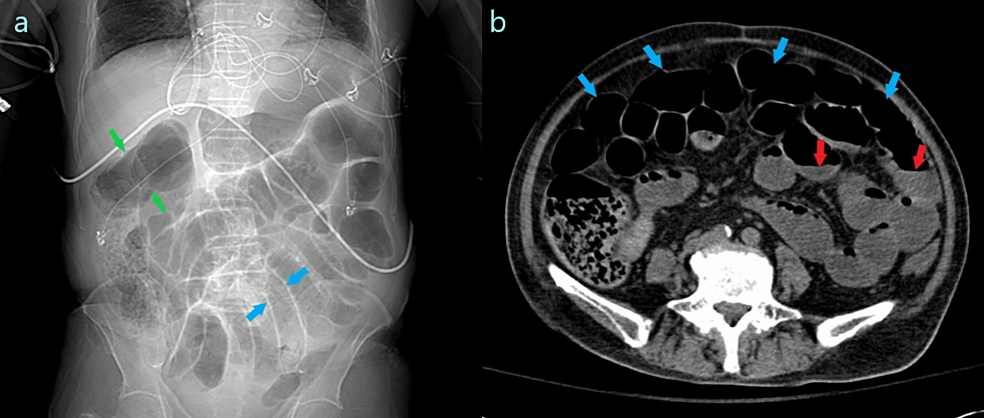
Computed tomography demonstrating ileus. (a) Scout image from computed tomography of the abdomen demonstrating diffuse gaseous distention of the large bowel (green arrows) and small bowel (blue arrows). (b) Axial computed tomography image through the abdomen demonstrating multiple loops of dilated small bowel (blue arrows) with air fluid levels (red arrows) and no transition point.

On admission to the hospital, the patient’s vital signs were within normal limits other than a heart rate of 111 beats per minute. Physical examination was notable for a markedly distended but soft and non-tender abdomen without rebound or guarding. Laboratories on admission were notable for a white blood cell count of 370/uL (normal 4,160-9,950/uL) with an absolute neutrophil count of 50/uL (normal 1,800-6,900/uL), hemoglobin of 12 g/dL (normal 13.5-17.1 g/dL), platelet count of 44,000/uL (normal 143,000-398,000/uL), glucose of 103 mg/dL (normal 65-99 mg/dL), bicarbonate of 13 mmol/L (normal 20-30 mmol/L), creatinine of 1.38 mg/dL (normal 0.6-1.3 mg/dL), blood urea nitrogen of 59 mg/dL (normal 7-22 mg/dL), aspartate transaminase of 317 U/L (normal 13-47 U/L), alanine transaminase of 328 U/L (normal 8-64 U/L), and alkaline phosphatase of 275 U/L (normal 37-113 U/L). Venous blood gas demonstrated a pH of 7.26 and COVID-19 polymerase chain reaction test returned positive. Lactate and beta-hydroxybutyrate levels were checked and were 25 mg/dL (normal 5-25 mg/dL) and 19.7 mg/dL (normal <3 mg/dL), respectively. Computed tomography of the abdomen and pelvis demonstrated an ileus (Figure 1).

**Figure 2:**
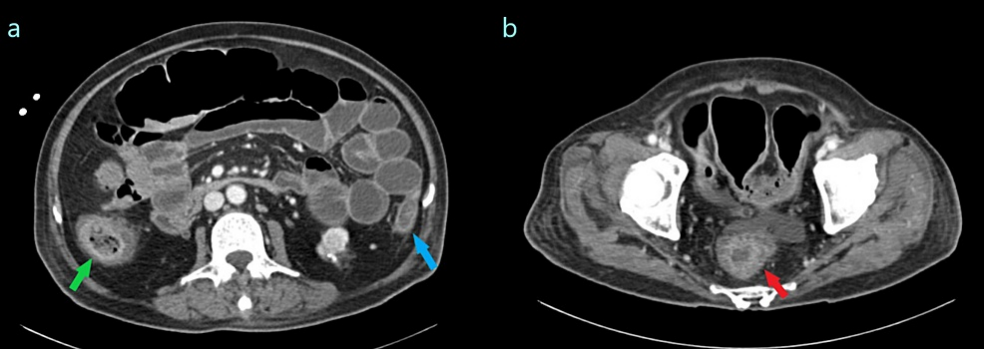
Computed tomography angiogram suggesting non-occlusive mesenteric ischemia. (a and b) Axial contrast-enhanced images through the abdomen demonstrate diffuse small bowel dilatation with colonic wall thickening predominantly affecting the watershed regions of the bowel despite patent vasculature. Green arrow: hepatic flexure. Blue arrow: splenic flexure. Red arrow: rectum.

Upon admission, there was concern for euglycemic DKA in the setting of dapagliflozin use as well as drug-induced agranulocytosis and liver injury secondary to triple therapy. Triple therapy medications as well as dapagliflozin were withheld, and the patient was started on a dextrose infusion as well as an insulin drip. Several hours after hospital admission, the patient developed shock with worsening acidosis and was transferred to the intensive care unit where he required vasopressors. A computed tomography angiogram of the abdomen and pelvis demonstrated findings suggestive of non-occlusive mesenteric ischemia (Figure 2).

After initiating treatment for euglycemic DKA, the patient's beta-hydroxybutyrate level decreased sequentially until it was not detectable. However, despite resolution of the patient's ketonemia, there was an ongoing metabolic acidosis. At this time, further history was obtained. The patient had previously been taking colchicine, which was removed from his medication list when he was recently started on triple therapy. However, the patient had overlapped ingestion of colchicine and clarithromycin for three days, starting one week prior to his acute presentation. The patient was diagnosed with colchicine toxicity as well as euglycemic DKA.

The patient was treated with supportive care, including filgrastim, blood and platelet transfusions, aggressive electrolyte and fluid balance management, bowel decompression via nasogastric tube, and bowel rest with the administration of total parenteral nutrition. The patient was also treated with broad-spectrum antibiotics, though no infectious source was ever found. The patient did not demonstrate any signs of hypoxic respiratory failure, so he did not receive any specific COVID-19 therapies during the hospital admission. The patient slowly improved with supportive therapies, but he had a prolonged hospital course, complicated by hospital-acquired pneumonia, urinary retention, recurrent hypotension, and recurrent ileus. The patient was ultimately discharged 26 days after admission. In the following several months, the patient had two admissions for aspiration pneumonia with respiratory failure. Given his recurrent aspiration pneumonias and his general ongoing functional decline, the patient ultimately elected to move forward with hospice care and passed away within six months from his initial presentation with colchicine toxicity.

## Discussion

Clarithromycin and colchicine should not be co-administered, as there is a high risk of colchicine toxicity. Colchicine is metabolized by the CYP3A4 system and is also a substrate for P-glycoprotein. Clarithromycin inhibits both the CYP3A4 system and P-glycoproteins, leading to elevated colchicine concentrations [5,6]. Other commonly used substances with similar properties that should not be co-administered with colchicine include erythromycin, itraconazole, ketoconazole, some protease inhibitors, and grapefruit juice [2]. These drug interactions have the potential to be fatal. In a retrospective study examining 88 patients who received colchicine and clarithromycin concomitantly, 10% of the patients died, and mortality was associated with longer overlapped therapy and renal impairment [7].

Colchicine toxicity presents in three clinical stages [2]. The first stage is in the initial 24 hours, during which the patient experiences gastrointestinal symptoms including nausea, vomiting, diarrhea, abdominal pain, hypovolemia, and leukocytosis. This first stage can clinically resemble acute gastroenteritis. The second stage generally occurs between one and seven days after ingestion and is marked by multiorgan failure, including respiratory failure, cardiac arrhythmias and arrest, encephalopathy, seizures, renal failure, liver failure, disseminated intravascular coagulation, bone marrow suppression, pancytopenia, hemolysis, metabolic derangements, myopathy, neuropathy, and secondary sepsis. The third stage occurs one to three weeks after ingestion and includes general recovery with possible rebound leukocytosis and alopecia.

Metabolic acidosis is a prominent feature of the second clinical stage of colchicine toxicity. The patient presented was recently prescribed dapagliflozin and had findings consistent with euglycemic DKA. Since it was not evident initially that the patient had been taking colchicine, he was initially diagnosed with a dapagliflozin-related euglycemic DKA in the setting of his poor oral intake. However, despite treatment for DKA, the patient had progressive clinical decline, which ultimately prompted further history gathering and led to the diagnosis of colchicine toxicity. This case highlights the importance of exploring a broad differential diagnosis for the various precipitating factors of DKA.

The diagnosis of colchicine toxicity was made based on the history of co-ingestion with clarithromycin and the patient's classic symptoms and signs. A plasma colchicine level was not obtained in the presented case as it was not readily available. Plasma colchicine levels are not necessarily helpful in the management of colchicine toxicity, as there is no established correlation with the severity of illness [2]. The diagnosis of euglycemic DKA was made based on the patient's history of ingestion of dapagliflozin and findings of metabolic acidosis and ketonemia. Ketonemia is not expected with acute colchicine toxicity. While ketonemia could also be due to starvation ketoacidosis or alcoholic ketoacidosis, given that the patient was taking dapagliflozin and did not have any significant history of alcohol use, the diagnosis of euglycemic DKA was favored.

The patient in the presented case also had findings suggestive of non-occlusive mesenteric ischemia. Non-occlusive mesenteric ischemia has been previously reported in another case of colchicine toxicity [8]. It remains unclear if colchicine toxicity directly causes non-occlusive mesenteric ischemia. However, non-occlusive mesenteric ischemia is associated with critical illness and multiorgan dysfunction, and cardiovascular disease and chronic renal insufficiency are known risk factors [9]. 

As demonstrated in the presented case, the treatment for colchicine toxicity is largely supportive, including aggressive electrolyte and fluid balance management as well as prevention of further morbidity [10]. Granulocyte colony-stimulating factor should be utilized for leukopenia. If the patient presents acutely after ingestion, removing any remaining colchicine from the gastrointestinal tract should be attempted with gastric lavage and activated charcoal.

Given the effect of colchicine toxicity on multiple organs, colchicine toxicity may initially appear to resemble other more commonly seen diagnoses such as sepsis and/or DKA. Thorough history gathering and a high index of suspicion by physicians and other providers are necessary for the diagnosis of colchicine toxicity. Given the lack of directed therapies to ameliorate toxicity, appropriate colchicine dosing and avoidance of interacting drugs are essential to the prevention of toxicity.

## Conclusions

Colchicine toxicity has been well described, but to our knowledge, we have presented the first case in the literature of a patient presenting with concomitant euglycemic DKA. In this case, the patient was first diagnosed with euglycemic DKA but, despite appropriate treatment, continued to have ongoing metabolic acidosis. Further history taking led to the diagnosis of colchicine toxicity. This case demonstrates the need for thorough history gathering and a high index of suspicion for the diagnosis of colchicine toxicity. Colchicine toxicity may initially present with or as other more commonly seen pathologies and can lead to multiorgan failure. Prevention of toxicity is key with appropriate colchicine dosing and avoidance of interacting medications.
